# Genomic Characterization of Colistin and Fluoroquinolone Resistance in Multidrug-Resistant *Escherichia coli* from Diseased Food-Producing Animals in Taiwan

**DOI:** 10.3390/antibiotics15080765

**Published:** 2026-08-10

**Authors:** Nan-Ling Kuan, Kuang-Sheng Yeh

**Affiliations:** 1 Biology Division, Veterinary Research Institute, Ministry of Agriculture, Tamsui, New Taipei City 251018, Taiwan; nlkuan@mail.nvri.gov.tw; 2 Department of Veterinary Medicine, School of Veterinary Medicine, National Taiwan University, Taipei 106319, Taiwan

**Keywords:** *Escherichia coli*, antimicrobial resistance, whole-genome sequencing, colistin resistance, fluoroquinolone resistance, *mcr* genes, One Health

## Abstract

**Background**: The global spread of antimicrobial resistance in *Escherichia coli* (*E. coli*) from food-producing animals is a critical concern within the One Health framework, particularly because of the potential transmission of clinically relevant resistance determinants across the animal–environment–human interface. Although extended-spectrum β-lactamase (ESBL)-producing *E. coli* strains have been widely characterized, the genomic mechanisms underlying resistance to the last-resort and critically important antimicrobials colistin and fluoroquinolones remain incompletely understood in animal-associated populations. This study characterized these resistance mechanisms in multidrug-resistant (MDR) *E. coli* from diseased food-producing animals. **Methods**: We used whole-genome sequencing (WGS) to characterize 77 MDR *E. coli* isolates from diseased livestock and poultry in Taiwan and analyzed the underlying resistance mechanisms and their co-occurrence. **Results**: Plasmid-mediated colistin resistance genes, including *mcr-1.1*, *mcr-3.1*, and *mcr-3.5*, were identified alongside chromosomal mutations, such as *pmrB* substitutions, indicating multiple evolutionary pathways to colistin resistance. Fluoroquinolone resistance was driven by both chromosomal mutations in quinolone resistance-determining regions and plasmid-mediated quinolone resistance genes, including *qnr* variants and *aac(6′)-Ib-cr*. Notably, the frequent co-occurrence of resistance determinants targeting multiple antimicrobial classes suggests the presence of mobile genetic elements facilitating horizontal gene transfer. **Conclusions**: Although a subset of isolates overlapped with those reported in our previous ESBL-focused study, the present work presents a comprehensive genomic dissection of resistance mechanisms beyond β-lactamases. The convergence of colistin, fluoroquinolone, and ESBL-associated resistance determinants within individual isolates highlights the potential role of food-producing animals as reservoirs of multidrug resistance, with potential implications for cross-sectoral transmission. These findings underscore the importance of integrated surveillance strategies addressing potential zoonotic transmission under the One Health framework.

## 1. Introduction

The global dissemination of antimicrobial resistance among *Escherichia coli* (*E. coli*) has become a major concern in both human and veterinary medicine [1]. In particular, multidrug-resistant (MDR) *E. coli* strains originating from food-producing animals are increasingly recognized as key reservoirs of resistance determinants that can disseminate through the food chain, the environment, and direct animal–human contact within a One Health framework [2,3].

Colistin and fluoroquinolones are critical antimicrobials in both human and veterinary clinical practice [1]. Colistin is considered a last-resort antibiotic for the treatment of infections caused by carbapenem-resistant Gram-negative bacteria. Notably, the emergence of plasmid-mediated colistin resistance genes, particularly *mcr* variants, has raised considerable global concern because of their high transferability and potential for rapid dissemination [4,5]. Additionally, fluoroquinolones are widely used in veterinary medicine, and resistance to these antibiotics arises primarily from chromosomal mutations in quinolone resistance-determining regions (QRDRs) as well as plasmid-mediated quinolone resistance (PMQR) genes such as *qnr* and *aac(6′)-Ib-cr* [6].

Food-producing animals are key reservoirs of both colistin- and fluoroquinolone-resistant *E. coli* [2]. Extended-spectrum β-lactamase (ESBL)-producing *E. coli* strains occur widely in livestock and poultry and thus play a vital role in antimicrobial resistance dissemination across animal, environmental and human sectors [7,8]. In our previous investigation, ESBL-producing *E. coli* isolated from diseased livestock and poultry in Taiwan exhibited a high prevalence of *bla*_CTX-M_ genes, particularly *bla*_CTX-M-55_, and demonstrated the capacity for horizontal gene transfer through conjugation [9]. However, that study primarily focused on phenotypic resistance and ESBL-related genes and did not address the genomic basis of resistance to other critical antimicrobials.

Whole-genome sequencing (WGS) enables comprehensive characterization of the resistome and elucidation of the genetic mechanisms underlying antimicrobial resistance [10,11]. WGS also facilitates the identification of both chromosomal mutations and horizontally acquired resistance genes as well as the analysis of clonal relationships and potential transmission pathways [10].

In the current study, we employed WGS to investigate the genetic determinants of colistin and fluoroquinolone resistance in MDR *E. coli* isolates obtained from diseased food-producing animals in Taiwan. A subset of isolates analyzed in this study was derived from our previous ESBL-focused investigation [9], and additional isolates were included to expand the dataset. This study (i) characterized the distribution of colistin resistance genes, including *mcr* variants; (ii) identified chromosomal and plasmid-mediated mechanisms associated with fluoroquinolone resistance; and (iii) analyzed the co-occurrence of resistance determinants within the broader resistome. Our findings are expected to provide crucial genomic insights into antimicrobial resistance in animal-derived *E. coli* and contribute to a better understanding of their potential impact within a One Health context.

## 2. Results

### 2.1. Genomic Overview and Phylogenetic Distribution

**Figure 1:**
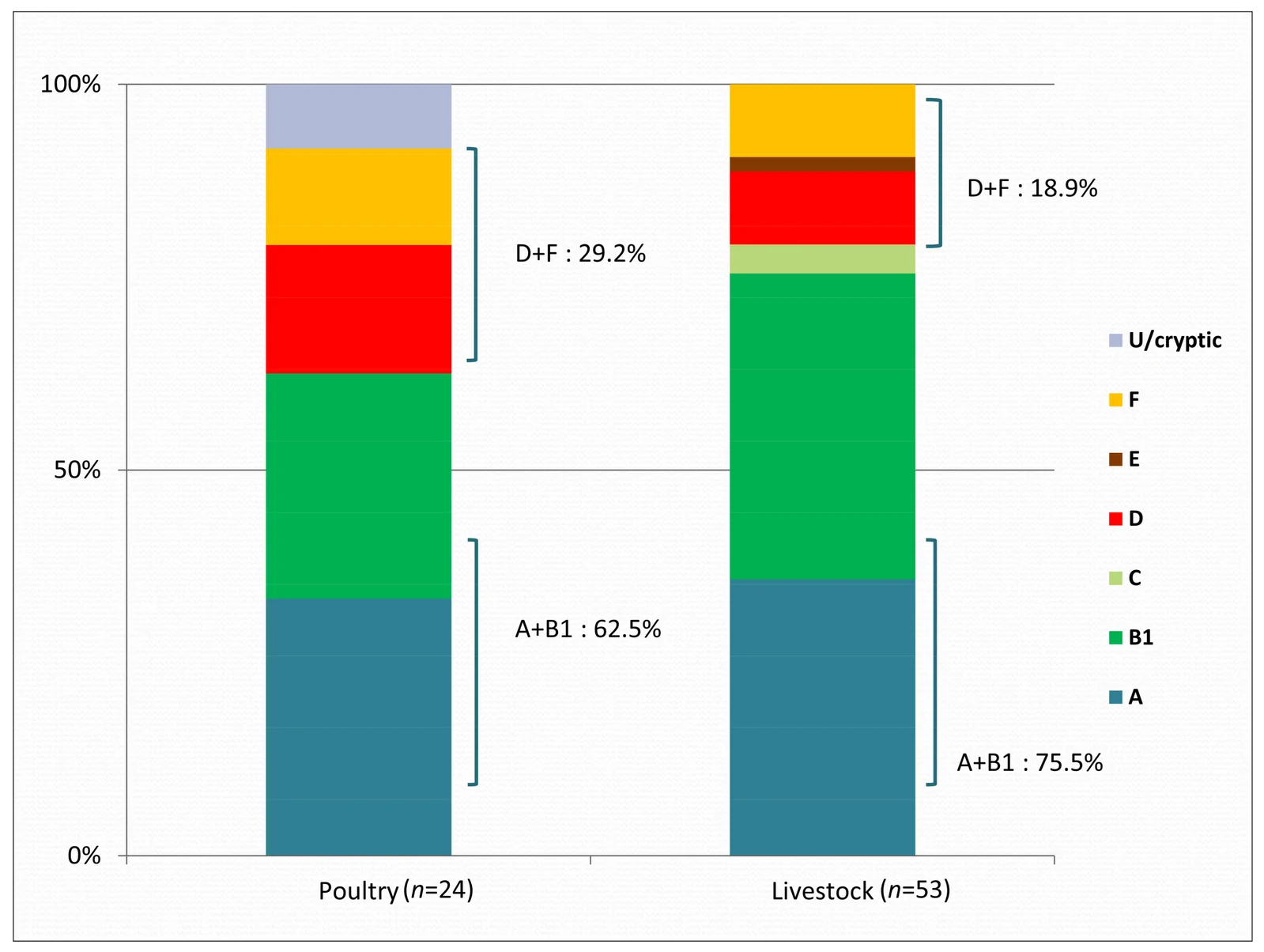
Phylogenetic distribution of 77 multidrug-resistant (MDR) *Escherichia coli* (*E. coli*) isolates from food-producing animals. Phylogroups were assigned using the updated Clermont scheme on the basis of whole-genome sequencing (WGS) data, and the phylogroups of the poultry (*n* = 24) and livestock (*n* = 53) isolates are presented separately. Phylogroups A and B1 predominated in both groups, whereas phylogroups D and F were more frequently observed among poultry isolates.

The updated Clermont phylogrouping scheme classifies *E. coli* into seven major phylogroups (A, B1, B2, C, D, E, and F) and cryptic clades, which differ in their evolutionary relationships and ecological characteristics. Phylogroups A and B1 are commonly associated with commensal strains, whereas phylogroups B2, D, and F are more frequently associated with extraintestinal pathogenic *E. coli* (ExPEC). Phylogroup E has been associated with certain intestinal pathogenic lineages, and cryptic clades represent genetically distinct *Escherichia* lineages [12]. In our study, phylogenetic analysis of the 77 MDR *E. coli* isolates based on the updated Clermont scheme revealed a heterogeneous phylogenetic distribution (Figure 1). Phylogroups A and B1 predominated in poultry and livestock isolates, whereas phylogroups D and F were more frequently observed among poultry isolates. No isolates belonged to phylogroup B2. Smaller proportions of isolates belonged to phylogroups C and E or were classified as cryptic lineages. Overall, the phylogroup distribution was dominated by lineages generally regarded as commensal or animal-adapted, with ExPEC-associated phylogroups being relatively infrequent.

### 2.2. Phylogenetic Distribution and Genotype–Phenotype Correlation of Colistin Resistance

**Figure 2:**
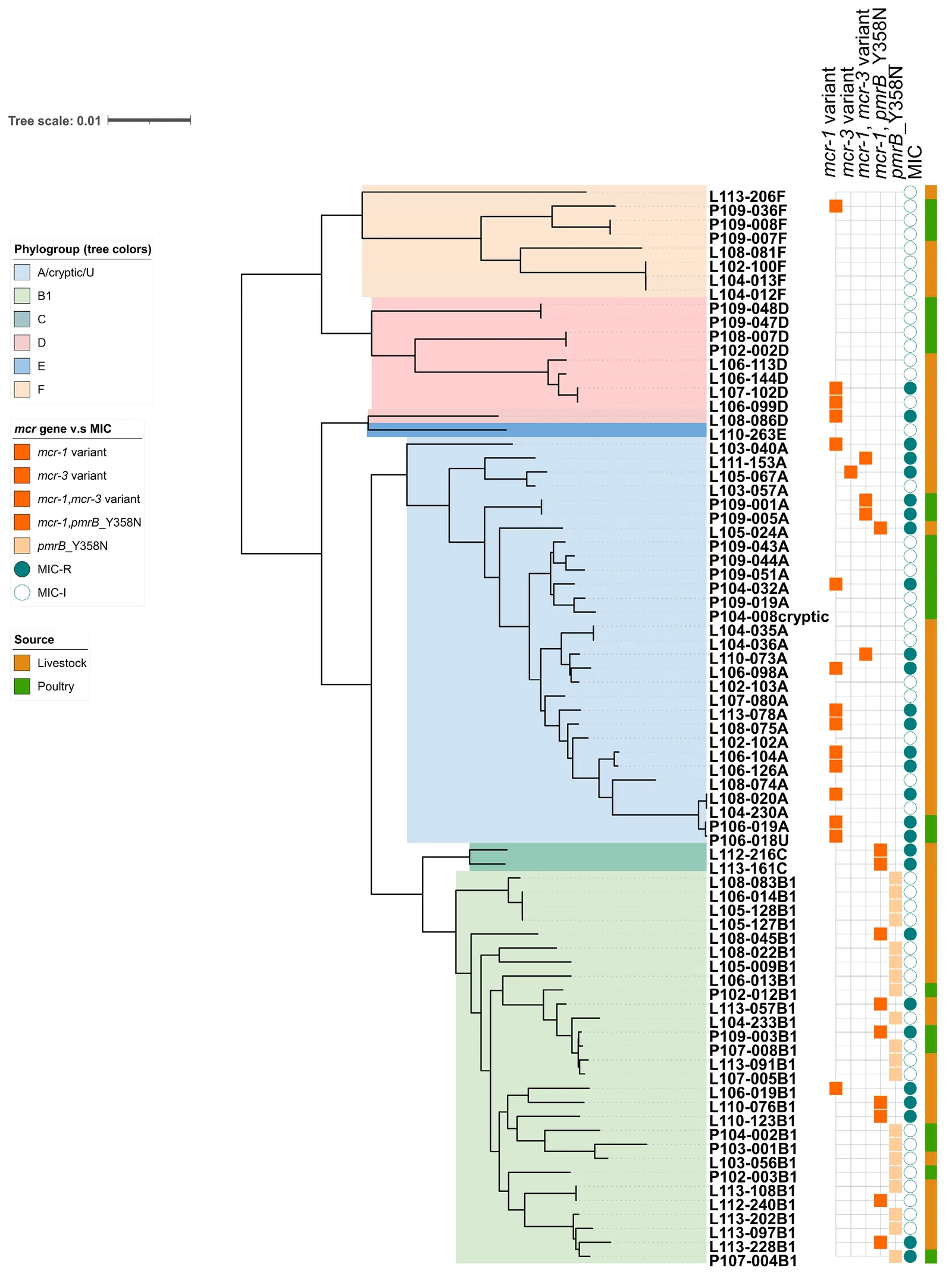
Phylogenetic distribution and genotype–phenotype correlation of colistin resistance in multidrug-resistant (MDR) *Escherichia coli* (*E. coli*). The phylogenetic tree of *E. coli* isolates was constructed on the basis of whole-genome sequencing (WGS) data, with branch colors indicating phylogroups according to the Clermont classification scheme (A/cryptic/U, B1, C, D, E, and F). Isolate identifiers are presented on the right. Colistin resistance determinants are indicated by colored squares, including *mcr-1* variants, *mcr-3* variants, combinations of *mcr-1* and *mcr-3*, and chromosomal mutations such as *pmrB* Y358N. Minimum inhibitory concentrations (MICs) are represented by circles. Filled green circles indicate resistant isolates, whereas open circles indicate intermediate isolates according to CLSI interpretive criteria. Poultry and livestock sources are represented using different colors. Most isolates carrying colistin resistance determinants exhibited elevated MICs and resistant or intermediate phenotypes. Resistance determinants were distributed across phylogenetic lineages rather than confined to a single clonal group.

Colistin resistance determinants were identified in 65% of the isolates (Figure 2). WGS revealed a diverse population structure among the MDR *E. coli* isolates that included multiple phylogroups, including A/cryptic/U, B1, C, D, E, and F. Among the identified resistance determinants, *mcr-1.1* was the most prevalent, followed by *mcr-3* variants. In addition to plasmid-mediated resistance, chromosomal mutations, including *pmrB* Y358N, were detected. Some isolates carried combinations of *mcr* genes and chromosomal mutations, indicating the coexistence of multiple resistance mechanisms.

Mapping resistance determinants onto the phylogenetic tree revealed that *mcr* genes and chromosomal mutations were distributed across phylogenetic lineages and that resistance determinants were identified in both poultry and livestock isolates, although *mcr*-associated resistance appeared more frequently in poultry isolates.

Comparison of genotypic and phenotypic data revealed that most isolates harboring colistin resistance determinants exhibited elevated minimum inhibitory concentration (MIC) values and resistant or intermediate phenotypes. Nevertheless, a few genotype–phenotype discrepancies remained.

### 2.3. Fluoroquinolone Resistance Mechanisms and Genotype–Phenotype Correlation

**Figure 3:**
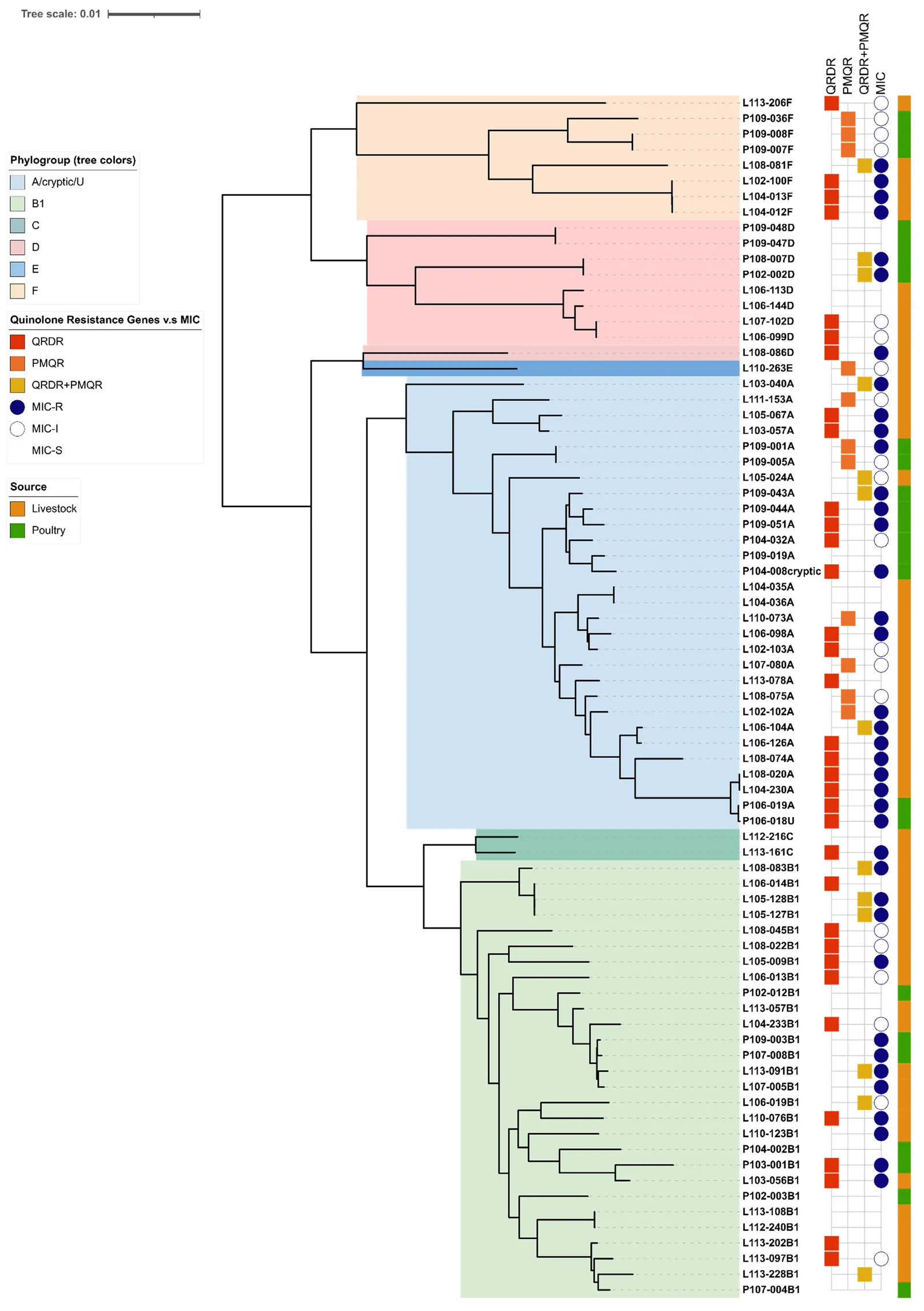
Phylogenetic distribution and genotype–phenotype correlation of fluoroquinolone resistance in multidrug-resistant (MDR) *Escherichia coli* (*E. coli*). The phylogenetic tree of *E. coli* isolates was constructed on the basis of whole-genome sequencing (WGS) data, and the branch colors indicate phylogroups based on the Clermont classification scheme (A/cryptic/U, B1, C, D, E, and F). Isolate identifiers are displayed on the right. Fluoroquinolone resistance mechanisms are indicated by colored squares, including chromosomal mutations in quinolone resistance-determining regions (QRDR), plasmid-mediated quinolone resistance (PMQR), and their combination (QRDR + PMQR). Minimum inhibitory concentrations (MICs) are represented by circles, with filled blue circles indicating resistant isolates, open circles indicating intermediate isolates, and an absence of circles indicating susceptible isolates, with classification based on Clinical and Laboratory Standards Institute (CLSI) breakpoints. Livestock and poultry isolates are indicated using distinct colors. Resistance determinants were distributed across phylogenetic lineages. Overall, most resistance determinants corresponded well with phenotypic susceptibility profiles, although limited genotype–phenotype discrepancies were observed.

Fluoroquinolone susceptibility testing revealed variable resistance phenotypes, including susceptible (S), intermediate (I), and resistant (R) phenotypes (Figure 3). Phylogenetic analysis demonstrated that the isolates belonged to multiple phylogroups, indicating diverse genetic backgrounds. Fluoroquinolone resistance determinants were distributed across phylogenetic lineages rather than restricted to a specific clonal group. Genomic analysis identified both chromosomal mutations and plasmid-mediated determinants associated with fluoroquinolone resistance. Mutations in QRDRs, particularly in *gyrA* and *parC*, were frequently detected and were likely major contributors to high-level resistance. In addition, PMQR genes, including *qnr* variants and *aac(6′)-Ib-cr*, were widely observed. Multiple isolates harbored both QRDR mutations and PMQR genes, indicating the coexistence of combined resistance mechanisms. Comparison between genotypic and phenotypic data demonstrated that such isolates generally exhibited elevated MICs and phenotypic resistance. However, genotype–phenotype discrepancies were also observed. Some isolates carrying PMQR genes exhibited susceptible or intermediate phenotypes, and others lacking detectable PMQR genes still exhibited elevated MICs. Furthermore, isolates from both livestock and poultry sources were distributed across phylogenetic groups, and resistance determinants were identified in isolates from both sources, indicating that fluoroquinolone resistance is not confined to a specific host-associated lineage.

These findings suggest that fluoroquinolone resistance in this collection is mediated by multiple mechanisms, including chromosomal mutations and plasmid-mediated genes, and that their combined effects contribute to the observed resistance phenotypes.

### 2.4. Resistome Profiling and Co-Occurrence of Resistance Determinants

Comprehensive resistome analysis demonstrated that all MDR *E. coli* isolates harbored multiple antimicrobial resistance genes, reflecting extensive resistance diversity within the studied population. Commonly detected determinants included *tetA* and *tetB* (tetracycline resistance); *sul1*, *sul2*, and *sul3* (sulfonamide resistance); various aminoglycoside resistance genes (*aac*, *aadA*, and *aph* variants); and β-lactamase genes such as *bla*_CTX-M-55_ and *bla*_CMY-2_.

Most isolates carried at least eight resistance genes, whereas several isolates harbored substantially larger numbers of resistance determinants. Genes conferring resistance to multiple antimicrobial classes, including colistin, fluoroquinolones, β-lactams, tetracyclines, sulfonamides, and aminoglycosides, were frequently identified within the same isolate. Notably, colistin resistance genes (*mcr* variants) frequently co-occurred with β-lactamase genes and fluoroquinolone resistance determinants, including QRDR mutations and PMQR genes. This phenomenon was observed in both poultry and livestock isolates and across phylogenetic lineages. These findings indicate extensive accumulation and co-localization of antimicrobial resistance determinants within individual isolates, suggesting that multidrug resistance platforms are potentially associated with mobile genetic elements, such as plasmids.

## 3. Discussion

Our findings provide a genomic characterization of MDR *E. coli* from diseased food-producing animals in Taiwan, revealing substantial diversity in phylogenetic backgrounds and antimicrobial resistance determinants. The coexistence of multiple resistance mechanisms across genetically diverse isolates highlights the complexity of antimicrobial resistance in food-animal-associated *E. coli* populations.

### 3.1. Phylogenetic Diversity and Population Structure

WGS revealed substantial genetic diversity among MDR *E. coli* isolates, with strains distributed across phylogroups, including A/cryptic/U, B1, C, D, E, and F. *E. coli* phylogroups differ in terms of their ecological distribution and host association, with phylogroups A and B1 being commonly associated with commensal and animal-adapted strains [12,13]. We observed that isolates from both poultry and livestock sources were broadly dispersed across phylogenetic groups, indicating that population structures were heterogeneous and that antimicrobial resistance in this population may not be driven solely by expansion of a single clone; it may also involve dissemination of resistance elements across unrelated lineages [14]. Similar observations were reported in food-animal-associated *E. coli* populations, in which antimicrobial resistance determinants were distributed across genetically diverse lineages rather than restricted to specific phylogenetic groups [15].

Phylogroups A and B1 were predominant in the present study, accounting for nearly three-quarters of all isolates. These phylogroups are generally regarded as commensal or animal-adapted lineages and are commonly recovered from the intestinal microbiota of healthy livestock and poultry [12,13]. No isolates belonged to phylogroup B2, which is commonly associated with ExPEC strains responsible for urinary tract infections, septicemia, and other invasive infections in humans and companion animals [13]. This finding indicates that multidrug resistance in this collection was primarily maintained within commensal or animal-adapted lineages rather than classical ExPEC backgrounds. Although phylogroups A and B1 are generally considered less virulent than ExPEC-associated lineages, they may nonetheless serve as important reservoirs of clinically relevant antimicrobial resistance genes. The widespread distribution of resistance determinants across genetically diverse lineages suggests that horizontal acquisition of mobile genetic elements, including plasmids carrying *mcr*, ESBL, and PMQR genes, may play an important role in antimicrobial resistance dissemination. Such populations may therefore serve as reservoirs from which resistance determinants can spread to pathogenic *E. coli* and other members of the order Enterobacterales within the One Health continuum. In summary, the MDR *E. coli* population in food-producing animals exhibited extensive phylogenetic diversity as well as widespread dissemination of mobile resistance determinants. This genomic background provides an essential framework for interpreting the colistin and fluoroquinolone resistance mechanisms discussed in the following sections.

### 3.2. Colistin Resistance Mechanisms and Genotype–Phenotype Correlation

A key finding of this study is the identification of both plasmid-mediated and chromosomal mechanisms contributing to resistance against critical antimicrobials in food-animal-associated *E. coli*. The detection of *mcr* variants, including *mcr-1.1* and *mcr-3* subtypes, confirms that transferable colistin resistance was present in these isolates. Since *mcr-1* was first described, plasmid-mediated colistin resistance has raised substantial concern among public health authorities and the scientific community given its potential for horizontal dissemination across bacterial species and ecological niches [4,16]. The identification of *pmrB* mutations in the present study implies that chromosomal adaptations may also contribute to colistin resistance. Modifications in two-component regulatory systems, including *pmrAB* and *phoPQ*, may alter lipid A modification pathways and reduce colistin susceptibility [17]. The coexistence of both resistance mechanisms within the same population demonstrates the complexity of colistin resistance and suggests that multiple resistance pathways contribute to the persistence and dissemination of colistin resistance under antimicrobial selective pressure.

Overall, resistance genotype showed good agreement with phenotypic susceptibility results, supporting the reliability of genomic prediction of colistin resistance in most isolates. Nevertheless, a few genotype–phenotype discrepancies were observed. Some *mcr*-positive isolates exhibited relatively low MICs, whereas certain isolates lacking detectable resistance determinants exhibited elevated MICs. Similar observations have been reported previously and may be explained by differences in gene expression, plasmid copy number, or regulatory mechanisms or the contribution of chromosomal mutations not fully captured by current resistance databases [17]. In addition, colistin susceptibility testing itself remains technically challenging because of factors such as heteroresistance and adsorption of colistin to laboratory materials, which contribute to MIC variability [18].

In summary, the current findings demonstrate that colistin resistance in MDR *E. coli* is mediated by diverse mechanisms involving both transferable resistance genes and chromosomal adaptations. Integrated genomic and phenotypic analysis remains essential for accurate colistin resistance profiling.

### 3.3. Fluoroquinolone Resistance Mechanisms

Fluoroquinolone resistance was primarily associated with chromosomal mutations in QRDRs, particularly in *gyrA* and *parC*, which are well-established determinants of high-level resistance and act by reducing drug–target binding [19]. The high prevalence of QRDR mutations among the isolates in this study suggests that chromosomal adaptation plays a major role in the development of fluoroquinolone resistance in *E. coli* from food-producing animals. PMQR genes, including *qnr* variants and *aac(6′)-Ib-cr*, were also identified; although they typically confer only low-level resistance, they may facilitate the selection and maintenance of higher-level resistance by enabling bacterial survival under subinhibitory antimicrobial concentrations [20]. The co-occurrence of PMQR genes and QRDR mutations observed in this study is particularly concerning because it suggests the accumulation of complementary resistance mechanisms within individual isolates, which promotes the persistence and dissemination of resistant strains under antimicrobial selective pressure.

In this study, resistance determinants were broadly distributed across phylogenetic backgrounds and animal sources rather than being restricted to specific lineages, implying that both clonal expansion and dissemination of resistance determinants may contribute to the spread of resistance in food-animal populations. We also observed discrepancies between genotypic and phenotypic resistance: some isolates harboring PMQR genes exhibited susceptible or intermediate phenotypes, whereas others lacking detectable PMQR genes still exhibited elevated MICs. These findings may be explained by the limited contribution of PMQR genes alone, cumulative effects of multiple QRDR mutations, alterations in efflux systems, and additional resistance mechanisms or regulatory pathways not fully represented in current resistance databases [19,20].

The widespread occurrence of QRDR mutations and PMQR genes among isolates from food-producing animals further indicates the critical role of these environments as reservoirs for fluoroquinolone resistance, likely reflecting the continued antimicrobial selective pressure exerted by the extensive use of fluoroquinolones in veterinary medicine [20]. Studies have highlighted the role of food-animal production systems in the emergence and maintenance of antimicrobial resistance determinants [21].

### 3.4. Integration of Resistance Determinants and Resistome Profiles

Our results demonstrate a high frequency of co-occurrence of resistance determinants across antimicrobial classes, including colistin, fluoroquinolones, and β-lactams. Such co-localization strongly suggests the involvement of mobile genetic elements, such as plasmids, which may serve as platforms for the accumulation and transmission of resistance genes [14]. This finding is consistent with the concept that food-producing animals can act as reservoirs of multidrug resistance, with potential implications for transmission along the food chain or through environmental pathways [21].

### 3.5. One Health Implications and Study Context

Although several analyzed isolates were also included in our previous ESBL-focused investigation [9], the present work can be considered a substantial complementary and mechanistically focused extension of our previous findings because of the application of WGS to characterize resistance mechanisms associated with critical antimicrobials. The integration of resistome profiling and genomic analyses provided additional insights into resistance architecture that could not be resolved using conventional phenotypic approaches.

The detection of transferable colistin resistance genes and multidrug resistance profiles in animal-derived *E. coli* raises crucial concerns regarding the potential for cross-sectoral transmission [22]. Although we did not investigate direct transmission pathways, the genomic features identified, including the presence of mobile resistance determinants, suggest that these isolates may serve as reservoirs of antimicrobial resistance with the potential for cross-sectoral dissemination. Continued genomic surveillance integrating animal, human, and environmental isolates will be essential to clarify potential transmission pathways within the One Health framework and to support future risk assessment and mitigation strategies.

### 3.6. Limitations

This study has several limitations. First, the isolates were obtained from diseased animals, and therefore, they may not fully represent the broader population of commensal *E. coli* in food-producing animals. Second, functional validation of resistance mechanisms, such as expression analysis or conjugation assays specific to colistin and fluoroquinolone resistance genes, was not performed. Third, although the genomic data suggest the involvement of mobile genetic elements, detailed plasmid characterization was beyond the scope of this study. Future investigations should incorporate long-read sequencing and functional assays to elucidate the mobility and transmission potential of these resistance determinants.

## 4. Materials and Methods

### 4.1. Bacterial Isolates and Identification

We included 77 MDR *E. coli* isolates recovered from diseased food-producing animals through routine diagnostic submissions to the Veterinary Research Institute, Ministry of Agriculture, Taiwan, between 2013 and 2024. The collection comprised 24 poultry isolates, including chickens (*n* = 10), ducks (*n* = 5), geese (*n* = 6), and turkeys (*n* = 3), and 53 livestock isolates, including pigs (*n* = 33), cattle (*n* = 11), and goats (*n* = 9). The poultry isolates were recovered from the internal organs of birds diagnosed with colibacillosis, whereas the livestock isolates were isolated from fecal swabs collected from animals with diarrhea. A subset of the isolates was included in our previous investigation of ESBL-producing *E. coli* [9], and additional isolates were incorporated in the present study to expand the dataset for whole-genome analysis. For bacterial isolation, clinical specimens were inoculated onto 5% sheep blood agar and MacConkey agar (Becton, Dickinson and Company [BD], Franklin Lakes, NJ, USA) and incubated aerobically at 37 °C for 24 h. Lactose-fermenting colonies with morphology consistent with *E. coli* on MacConkey agar (BD) were selected and subcultured to obtain pure isolates. Species identification was subsequently confirmed using the VITEK^®^ 2 Compact automated identification system (bioMérieux, Marcy-l’Étoile, France) with GN identification cards.

### 4.2. Antimicrobial Susceptibility Testing

Antimicrobial susceptibility testing was performed for all isolates in accordance with Clinical and Laboratory Standards Institute (CLSI) guidelines [23]. Colistin susceptibility was evaluated using the CLSI-approved colistin broth disk elution method, and broth microdilution was performed for comparison. Colistin susceptibility categories were interpreted according to CLSI guidelines. Because CLSI does not define a susceptible category for colistin, only intermediate and resistant categories are presented throughout this study. All colistin susceptibility testing was conducted using cation-adjusted Mueller–Hinton broth (CA-MHB) (BD). Final colistin concentrations of 1, 2, and 4 μg/mL were achieved by aseptically adding 10-μg colistin sulfate disks (BD) to tubes containing 10 mL of CA-MHB, with one, two, and four disks per tube, respectively. A control tube without colistin disks (0 μg/mL) was also included. Susceptibility testing for enrofloxacin (a fluoroquinolone) was performed using the Sensititre system (Thermo Fisher Scientific, Waltham, MA, USA) with BOPO6F or AVIAN1F panels. *E. coli* ATCC 25922 was used as a quality control strain for all susceptibility testing, and *mcr-1*-positive *E. coli* NCTC 13846 was additionally included as a quality control strain for colistin susceptibility testing.

### 4.3. Genomic DNA Extraction and WGS

Genomic DNA was extracted from overnight cultures using the PureLink Genomic DNA Mini Kit (Thermo Fisher Scientific), according to the manufacturer’s instructions. Libraries were prepared using the Illumina DNA Prep kit (Illumina, San Diego, CA, USA) and sequenced on an Illumina MiSeq platform using a MiSeq Reagent Kit v2 (Illumina) (500 cycles), generating paired-end 2 × 250 bp reads. Raw paired-end reads were uploaded to EnteroBase (warwick.ac.uk), where de novo genome assembly was performed using its integrated quality-controlled pipeline [24,25]. The resulting assemblies were used for downstream phylogrouping, resistome profiling, and phylogenetic analysis. The raw sequence reads have been deposited in the NCBI Sequence Read Archive (BioProject accession number PRJNA1453210).

### 4.4. In Silico Phylogrouping and Antibiotic Resistance Gene Identification

In silico classification of *E. coli* phylogroups was performed using EnteroBase according to the Clermont phylogenetic scheme [24]. Acquired antimicrobial resistance genes and chromosomal point mutations, particularly colistin resistance determinants (*mcr* variants and *pmrB* mutations) and fluoroquinolone resistance determinants (QRDR mutations in *gyrA* and *parC*), were identified using AMRFinderPlus (v3.11.26) [26,27].

### 4.5. Phylogenetic Tree Construction and Visualization

To evaluate the genomic relationships among the multidrug-resistant isolates, phylogenetic analysis was performed using Hierarchical Clustering (HierCC) based on core-genome genetic distances within EnteroBase [28]. The resulting phylogenetic tree was exported, visualized, and annotated using iTOL v6 to integrate genomic lineages with resistance genotypes and phenotypic MICs [29].

## 5. Conclusions

This study provides a comprehensive genomic characterization of colistin and fluoroquinolone resistance in MDR *E. coli* isolates from diseased food-producing animals in Taiwan. The identification of both plasmid-mediated (*mcr* variants) and chromosomal resistance mechanisms, together with the frequent co-occurrence of resistance determinants across antimicrobial classes, highlights the complex genetic architecture underlying antimicrobial resistance in animal-associated *E. coli*.

The convergence of resistance to critical antimicrobials, including colistin, fluoroquinolones, and β-lactams, within individual isolates underscores the potential role of food-producing animals as reservoirs of multidrug resistance and raises concerns regarding the persistence and dissemination of antimicrobial resistance within and beyond animal populations. Our results emphasize the importance of genomic surveillance for monitoring antimicrobial resistance and provide valuable insights into resistance mechanisms relevant to potential zoonotic transmission within the One Health framework. Further studies integrating plasmid characterization and transmission dynamics are warranted to elucidate the mobility and spread of resistance determinants.

## Data Availability

Whole-genome sequencing data have been deposited in the NCBI Sequence Read Archive under BioProject accession number PRJNA1453210. All other relevant data are included within the manuscript.
